# Advancing Environmental Health Literacy: Validated Scales of General Environmental Health and Environmental Media-Specific Knowledge, Attitudes and Behaviors

**DOI:** 10.3390/ijerph16214157

**Published:** 2019-10-28

**Authors:** Maureen Y. Lichtveld, Hannah H. Covert, Mya Sherman, Arti Shankar, Jeffrey K. Wickliffe, Cecilia S. Alcala

**Affiliations:** 1 Department of Environmental Health Sciences, School of Public Health and Tropical Medicine, Tulane University, New Orleans, LA 70112, USA; mlichtve@tulane.edu (M.Y.L.); msherman1@tulane.edu (M.S.); jwicklif@tulane.edu (J.K.W.); calcala@tulane.edu (C.S.A.); 2 Department of Biostatistics and Data Science, School of Public Health and Tropical Medicine, Tulane University, New Orleans, LA 70112, USA; sarti@tulane.edu

**Keywords:** environmental health literacy, scale development, community–academic partnerships, water, food, air, knowledge, attitudes, behaviors

## Abstract

Environmental health literacy (EHL) involves understanding and using environmental information to make decisions about health. This study developed a validated survey instrument with four scales for assessing media-specific (i.e., air, food, water) and general EHL. The four scales were created as follows: 1) item generation: environmental health scientists and statisticians developed an initial set of items in three domains: knowledge, attitudes, and behaviors; 2) item review: items were reviewed for face validity; 3) validation: 174 public health students, the exploratory sample, and 98 community members, the test sample, validated the scales. The scales’ factor structure was based on exploratory factor analysis (EFA) and model fit was assessed through confirmatory factor analysis (CFA). For each scale, the final EFA resulted in an independent three-factor solution for knowledge, attitudes, and behaviors. Good fit for the three-factor structure was observed. Model fit for CFA was generally confirmed with fit indices. The scales showed internal consistency with Cronbach’s alpha from 0.63 to 0.70. The 42-item instrument represents an important contribution towards assessing EHL and is designed to enable meaningful engagement between researchers and community members about environmental health. The intended outcome is sustained community–academic partnerships benefiting research design, implementation, translation, dissemination, and community action.

## 1. Introduction

Environmental health literacy (EHL) refers to the understanding and use of information about potentially harmful environmental exposures and how those exposures influence health [1]. EHL encompasses a range of knowledge, attitudes, and behaviors that influence how individuals and communities use environmental information in making decisions about health [2]. EHL is often viewed as a dynamic process through which individuals and communities are increasingly able to apply a more nuanced understanding of environmental health (EH) risks, exposures, outcomes, and strategies to reduce adverse environmental exposures and promote health [1,3]. Given the diversity of potential environmental exposures, it is also important to note that EHL is often specific to personal and community context and the contaminated environmental media involved. The term “environmental media” refers to parts of the environment, such as soil, water, air, plants and animals, that can contain contaminants, potentially resulting in individual or community exposure. EHL may be increased through multiple strategies [3]. Often, the desired outcome of addressing EHL is to increase knowledge and promote action among individuals and communities, especially in situations where past exposures to hazardous substances may lead to both current and future adverse health effects [2].

Recently, the literature has emphasized the importance of information-seeking and decision-making skills that raise awareness and knowledge of EH and could result in health-protective actions [1,4,5,6]. There is also recognition that the sources of environmental exposures are often outside an individual’s control and that health disparities can heighten community concerns [7]. Furthermore, socioeconomic and contextual factors influence EHL, including language, education, community networks, and news media [3,8]. Accordingly, it is necessary to address EH concerns utilizing a culturally competent, interdisciplinary, and context-specific approach [3,5,9]. Gray [1] presents three interrelated dimensions of EHL, including awareness and understanding, skills and self-efficacy to reduce harmful environmental exposures and make decisions that protect health, and community change to reduce or remove environmental exposures that are harmful to health. A range of activities are required to improve EHL across these dimensions. It is thus important to address EHL at both individual and community levels, ensuring that baseline awareness and knowledge is coupled with skills to ignite actions that promote health and reduce adverse exposure [2].

The field of EHL emerged in the 1970s from the American movements to promote literacy, including health literacy, science literacy, and environmental literacy, as well as risk communication and environmental justice [2,7]. Since 2014, there have been increasing efforts to define, measure, and improve EHL [1]. Some progress has been made in measuring EHL [8,10], with most studies 1) describing the perceived relationship between environmental exposures and health outcomes; 2) focusing on a specific exposure (e.g., pesticides, lead in drinking water, arsenic in soil); 3) assessing the impacts of interventions, and/or 4) examining results dissemination of biomonitoring studies [1]. For example, in a systematic literature review of 31 articles related to EHL, Gray [1] highlighted numerous studies that employed pre-/post-assessments to measure changes in EHL in response to an intervention or environmental exposure at both individual and community levels. Many of the studies utilized community-based participatory research approaches and examined community participation in results dissemination for biomonitoring studies [4,11,12]. Several of these “report-back” studies concluded that engagement in research can advance participants’ EHL [1,11,13].

A validated scale that measures individual EHL is a foundational step to improving EHL at both individual and community levels [3,14]. Standardized measurement of EHL has the potential to inform needs assessments, measure national EHL trends, guide policy and planning, and assess the impact of educational initiatives and other interventions that aim to improve EHL [3,13,14]. While several EHL tools exist, few have been validated [1]. To date, there are only two peer-reviewed studies [8,10] focused on the development and validation of tools to measure EH knowledge, behaviors, and attitudes. Dixon and colleagues [8] validated the Environmental Health Engagement Profile, which examines how individuals engage with EH issues, including their experience with EH hazards, assumptions of risks, and actions taken both individually or collectively. Although this study utilized a systematic process to generate the EHL scale, it was only validated via phone with community members located in New Haven, Connecticut, and statistical analyses lacked confirmatory factor analysis. Ratnapradipa et al. [14] used a modified Delphi technique to develop and validate core EH categories and corresponding topics, creating an assessment tool with 443 potential questions. Given the number of potential items included in this scale, the researchers were unable to validate the entire survey with community members, instead receiving validation on topic-specific survey sections through focus groups [10]. Additionally, feedback from participants highlighted the difficulty of creating a general EHL survey due to the context-specific and technical nature of some EH issues, highlighting the importance of validating a general EHL scale across geographic areas. While these studies represent advancements in the field, the currently published EHL scales lack environmental media-specific assessments of knowledge, attitudes, and behaviors and have not been validated with community members in more than one geographic area.

To fill this gap, this study aims to develop a validated survey instrument to assess an individual’s knowledge, attitudes and behaviors related to EHL. The design reflects the multidisciplinary nature of EH, as evidenced by the creation of scales encompassing both general EH and three environmental media; food, air, and water. These media were deliberately selected since individuals have daily contact with them and contamination of these media is often the focus of community concerns. The four scales were validated with distinct samples in two geographic areas using advanced multivariate statistical methods, including exploratory and confirmatory factor analyses. Using the scales in community-based participatory research (CBPR) can strengthen community engagement, research translation, and health promotion, ultimately advancing the implementation of environmental epidemiological studies.

## 2. Materials and Methods 

### 2.1. Item Generation

A committee of six faculty and research staff from Tulane University’s Department of Environmental Health Sciences and Department of Biostatistics and Data Science developed an initial set of items for the Air, Water, Food, and General EH scales. Items were generated in three domains—knowledge, attitudes, and behaviors. Knowledge was defined as facts and information pertaining to EH gained through experience or education. Attitudes were defined as settled ways of thinking or feeling about EH. Behaviors were defined as how individuals act in response to EH concerns. Items consist of declarative statements, most written in the first person, with Likert-type scales for response. Knowledge and attitude items have a five-point agreement scale of strongly agree (5), agree (4), I don’t know (3), disagree (2), and strongly disagree (1). Behavior items have a five-point frequency scale of always (5), often (4), sometimes, (3), hardly ever (2), and never (1).

The committee’s work was informed by existing survey instruments and peer-reviewed literature related to the three environmental media and general EHL. Particular attention was paid to locating existing items that dealt with at least one of the five elements of the exposure pathway—source of exposure, environmental media and transport mechanism, point of exposure, route of exposure, and receptor population. Items were initially selected and adapted from several sources [8,10,15,16,17,18,19,20]. Each item was then categorized as knowledge, attitude, or behavior. As a group, the committee revised the list of items for a 5th grade literacy level and clear wording, discarded items with irrelevant or repeated content, developed new items, and recategorized some items by domain. Ultimately, the committee generated 126 items across the four scales, with 33 items for the Air scale, 36 items for Water, 23 items for Food, and 34 items for General EH. 

### 2.2. Item Review

The items developed by the committee were then reviewed for face validity by three faculty experts [21] from Tulane University’s Department of Environmental Health Sciences. The experts were not part of the committee that generated items for the scales. Each expert individually reviewed a paper or electronic copy of the scales and provided feedback in writing. Items with 100% agreement from the experts were retained, while items with less than 100% agreement were modified as suggested by the expert(s). Four new items were added. After expert review, there were a total of 130 items, with 35 items for the Air scale, 38 items for Water, 23 items for Food, and 34 items for General EH.

### 2.3. Scale Validation

The four scales were validated through exploratory and confirmatory factor analyses. To conduct the factor analyses, the scales were administered to two separate samples; an exploratory sample of public health students and a test sample of community participants. Exploratory factor analysis was conducted on data collected from the public health students to identify the underlying factor structure for each of the four scales and to identify the items to retain for the final scales. The factor structure of the final scales was then confirmed on data from community participants using confirmatory factor analysis.

Participants were administered a paper-based version of the scales that took 15–30 minutes to complete. Tulane University’s Institutional Review Board approved the research protocol (Study #1051970) and informed consent was obtained from all participants before taking the survey. Responses were coded from one to five and entered in an Excel workbook (version 15.0, Microsoft, Redmond, WA, USA).

### 2.4. Exploratory Sample 

A total of 174 graduate and undergraduate public health students from Tulane University in New Orleans, Louisiana, served as the exploratory sample for the first set of scales (Table 1). Participants were recruited from three different public health courses, two graduate level courses and one undergraduate course.

### 2.5. Test Sample

A total of 98 community members from New Orleans, Louisiana, and Nashville, Tennessee, validated the factor structure of the final scales obtained through exploratory factor analysis (Table 1). Participants in New Orleans (n = 44) were recruited from the membership of a church, while those in Nashville (n = 54) were recruited with the assistance of the Office of Faculty Affairs and Development at Meharry Medical College. The communities in these two cities were selected due to their historic burden of health disparities and their successful long-term track record in CBPR.

### 2.6. Statistical Analysis Plan

Exploratory and confirmatory factor analyses were used to assess the psychometric properties of the four scales. Indicators of items and factors in the exploratory factor analysis (EFA) models were evaluated based on four criteria to determine items and factors to be retained. Factors were retained based on scree plots, amount of variance explained, and a chi-square test determining sufficiency of factors. Items were evaluated based on their factor loadings. Factor loadings more than 0.40 were categorized as fair, above 0.55 were categorized as good, and those more than 0.71 were categorized as excellent or high [22]. Based on the criterion that scales have a simple structure, retained items were required to have high loadings on a single factor. Items with cross loadings above 0.25 were excluded. Items without a high loading on any factor were also excluded from the analysis. Factors with less than two items were not considered for further analysis [23,24]. Factor loading of the final solution was used to evaluate the interpretability of the factors.

Maximum likelihood was used to estimate the parameters in the confirmatory factor analysis (CFA). Model fit of the CFA model was assessed using several tests including the chi-square test from the absolute fit index and the Root Mean Square Error of Approximation (RMSEA) from the parsimony index. A RMSEA cutoff value of 0.10 was established. Internal consistency and reliability of the adapted items for all scales was examined using Cronbach’s alpha. The cutoff reliability was set at 0.70 [25]. All study hypotheses were tested at 5% significance and all analyses were conducted using the Statistical Analysis System (SAS) software (version 9.4, SAS Institute, Cary, NC, USA). 

## 3. Results

### 3.1. Air Scale

EFA was conducted to examine the underlying factor structure of the initial 33-item Air scale. Three iterations of EFA were needed to reach an interpretable factor solution. The third and final EFA resulted in a constrained three-factor solution with three knowledge, three attitude, and four behavior items (Table 2).

Items’ loading ranged from −0.65 to 0.64 and there were no cross loadings greater than −0.27. The chi-square test evaluating the underlying structure was not significant (χ^2^ = 17.73, *p* = 0.47) (Table 3), indicating that a three-factor solution was sufficient. The variability explained by each factor was adequate. The first factor, the knowledge subscale, explained 17.6% of the variance. The second factor, the attitudes subscale, explained 13.3% of the variance, and the third factor, the behaviors subscale, explained 13.8% of the variance.

The eigenvalues of the retained factors are shown in Table 4. The inter-factor correlations showed no significant associations between the three subscales; behaviors and attitudes (r = −0.12, *p* = 0.13); behaviors and knowledge (r = -0.05, *p* = 0.56); attitudes and knowledge (r = 0.06, *p* = 0.44). The final ten-item Air scale was subjected to CFA on data collected from the test sample of community participants (Table 3). Both the absolute index (χ^2^ = 41.23, *p* = 0.13) as well as the parsimony index (RMSEA = 0.05) indicated good fit of the items to their respective subscales. The Cronbach’s alpha for the Air scale was 0.70, indicating good internal consistency among the items.

### 3.2. Food Scale

Three iterations of EFA were needed to reach an interpretable factor solution for the initial 23-item Food scale. The third and final EFA resulted in a three-factor solution with two knowledge, five attitude, and two behavior items (Table 5). Items’ loading ranged from 0.39 to 0.99. A three-factor scale was considered sufficient (χ^2^ = 16.55, *p* = 0.17). The first factor, the knowledge subscale, explained 19.6% of the variance. The second factor, the attitudes subscale, explained 38.1% of the variance, and the third factor, the behaviors subscale, explained 2.6% of the variance. The eigenvalues of the retained factors are shown in Table 4. The inter-factor correlations between the attitudes and behaviors subscales (r = 0.18, *p* = 0.02) and the knowledge and behaviors subscales (r = 0.32, *p* <0.0001) were statistically significant. There were no significant associations between the attitudes and knowledge subscales (r = −0.02, *p* = 0.77). CFA on the nine-item Food scale based on data from the test sample showed that although the chi-square test (χ^2^ = 51.41, *p* <0.001) was significant, the RMSEA value of 0.11 was just above the cutoff of 0.10 (Table 3). Internal consistency of the scale (Cronbach’s alpha = 0.67) was slightly below the established cutoff of 0.70.

### 3.3. Water Scale

Two iterations of EFA were needed to reach an interpretable factor solution on the initial 36-item Water scale. The final EFA resulted in a 14-item scale with four knowledge, three attitude, and seven behavior items (Table 6). The chi-square test to evaluate the underlying factor structure was not significant (χ^2^ = 46.93, *p* = 0.67), indicating a three-factor solution was sufficient. The items had high loadings on their own factor ranging from −0.38 and 0.67 with no cross loadings greater than 0.26. The first factor, the knowledge subscale, explained 15.6% of the variance. The second factor, the attitudes subscale, explained 24% of the variance, while the third factor, the behaviors subscale, explained 26.4% of the variance. The eigenvalues of the retained factors are shown in Table 4. The inter-factor correlations showed no significant associations between the three subscales; behaviors and attitudes (r = 0.05, *p* = 0.45); behaviors and knowledge (r = 0.15, *p* = 0.16); attitudes and knowledge (r = −0.04, *p* = 0.57). CFA on the 14-item Water scale based on data from the test sample showed that while the chi-square test (χ^2^ = 119.77, *p* = 0.01) was statistically significant, the parsimony index indicated a good fit (RMSEA = 0.07) (Table 3). Internal consistency of the scale was slightly below the established cutoff of 0.70 (Cronbach’s alpha = 0.63).

### 3.4. General EH Scale 

Three iterations of EFA were completed to reach an interpretable solution for the initial 34-item General EH scale. The third and final EFA resulted in a three-factor solution with three knowledge, three attitude, and three behavior items (Table 7). Item loadings ranged from 0.26 to 0.99, and there were no cross loadings greater than 0.20. The underlying three-factor structure was sufficient (χ^2^ = 20.65, *p* = 0.30). The variability explained by the attitudes subscale was 17.6%, behaviors was 12.6%, and knowledge was 9.1%. The eigenvalues of the retained factors are shown in Table 4. The inter-factor correlations between the subscales was not significant; attitudes and behaviors (r = 0.09, *p* = 0.37); knowledge and behaviors (r = −0.04, *p* = 0.70); attitudes and knowledge (r = −0.04, *p* = 0.70). The CFA model showed good fit (χ^2^ = 28.56, *p* = 0.24 and RMSEA = 0.05) (Table 3). Internal consistency of the nine-item scale was measured using Cronbach’s alpha and was at the established cutoff of 0.70.

## 4. Discussion

The purpose of this study was to develop a psychometrically sound, multidimensional scale of independent factors to assess specific environmental media and general EHL. The survey instrument uses five-point Likert scales to assess respondents’ knowledge, attitudes, and behaviors related to EH issues. The four scales, with a total of 42 items, were developed using the results of exploratory and confirmatory factor analyses completed on data collected from distinct exploratory and test samples of public health students and community members, respectively.

The ten-item Air scale comprises a three-factor solution with three knowledge, three attitude, and four behavior items. All three subscales explain adequate amount of variability ranging from 13.3% to 17.6%. The Cronbach’s alpha for this scale was 0.70, indicating good internal consistency. The nine-item Food scale comprises a three-factor solution with five knowledge, two attitude, and two behavior items. All three subscales explain variance ranging from 2.6% to 38.1%. The Cronbach’s alpha for this scale was 0.67. The 14-item Water scale comprises a three-factor solution with five knowledge, two attitude, and two behavior items explaining 15.6–26.4% of the variance. Reliability of this scale was measured with a Cronbach’s alpha of 0.63. The nine-item General EH scale also resulted in a three-factor solution with three knowledge, three attitude, and three behavior items with the subscales explaining 9.1–17.6% of the variance. The Cronbach’s alpha for this scale was 0.70. Although all three factors of the four scales are distinct, they do have an implicit relationship, indicating some interdependence among them. Significant associations were found between the attitudes and knowledge subscales for all four scales. No significant associations were seen between any of the behaviors and knowledge subscales and the attitudes and behaviors subscales. All four scales show good fit statistics, with the Air scale and the General EH scale showing good absolute fit and all four scales showing good to adequate practical fit with RMSEA values ranging from 0.05 to 0.11.

The validated survey instrument developed in this study represents an important contribution towards evaluating individual EH knowledge, attitudes, and behaviors. The scales could be used to measure the effectiveness of a relevant intervention, however, the value of the scales does not lie in scoring or rating an individual’s EHL. Rather, the scales are best utilized in CBPR settings to a) gauge community partners’ general knowledge, attitudes, and behaviors related to EH, b) identify specific EH concerns among community partners, c) identify constraints that community partners might face in reducing adverse environmental exposures, d) inform research translation and dissemination, or e) serve as starting point for discussing EH issues and EHL among research partners. The four scales can also serve as a gap analysis to inform strategies to bolster community resilience [26] and tailor health promotion and education actions, further enabling community members to participate in both CBPR and environmental epidemiology studies.

Given the complex nature of many environmental exposures, it is important that community research partners have general knowledge of the completed exposure pathway and the biological plausibility of linking adverse health conditions to environmental exposures, using appropriate and well-accepted criteria [27]. For example, to advance effective community–academic partnerships in environmental health research, there must be common understanding that environmental exposure must precede disease for there to be a possible causal relationship between the two. Using the scales as a means for researchers and community members to meaningfully engage with each other about EH and EHL will lead to more sustained community–academic partnerships and build trust in the research findings. This, in turn, will benefit research translation, dissemination, and community action. The scales will be of interest to university-based research centers (e.g., Superfund Research Program Centers, Environmental Health Sciences Core Centers) and investigators engaged in environmental epidemiology studies. In addition to expanding the EH knowledge base, the scales can also strengthen CBPR and related educational products and curricula [28].

There are some limitations for this study. The exploratory sample cannot be classified as a probability sample since data were collected from students enrolled in public health degree programs. These participants, however, were appropriate for testing knowledge, attitudes, and behaviors associated with EH. Furthermore, the factor structure developed on the exploratory sample was confirmed on a different population of community participants. Other limitations include the lack of data to test the convergent and divergent validity of the scales, and the Cronbach’s alpha values of the Water scale (0.63) and the Food scale (0.67) just approach the recommended 0.70 minimum [25]. The expert review of the scales for face validity likely reflected the specific expertise of the reviewers within the broad field of EH. The four scales also reflect a content-driven approach to assessing individual EHL. This approach does not necessarily assess individual self-efficacy or account for contextual drivers of EHL. However, this validated instrument does consider behaviors and attitudes in addition to knowledge, and it represents an important vehicle for community-engaged research and action that addresses other dimensions of EHL. Considering the breadth of the EH field, the scales do not cover all possible environmental media (e.g., soil) or EH issues (e.g., radon, disasters, and climate change). However, the scales can serve as a blueprint for creating more detailed scales in additional EH domains. Finally, the survey items were intentionally written at a 5th grade literacy level to ensure that the validated instrument could be effectively administered to the general public. However, reducing complex EH topics to a brief set of items necessarily involved the loss of some nuance and detail in the instrument, which is an ongoing challenge in the EHL field [29].

## 5. Conclusions

This study presents the first validated survey instrument to assess an individual’s knowledge, attitudes, and behaviors related to EHL, contributing an important tool to environmental health research and practice. In contrast to previous EHL tools, the instrument presented in this study contains media-specific and general EH scales and was validated in its entirety with different audiences in two geographic areas through exploratory and confirmatory factor analyses. With only 42 items, it can be administered in a variety of community contexts and has the potential to advance the efficacy of EH interventions, build EH practice capacity, and bolster community engagement in CBPR projects. The four validated scales thus represent a critical step toward raising scientific and environmental health literacy.

## Figures and Tables

**Table 1:** Exploratory and test sample demographics.

Characteristic	Exploratory Sample Total, n (%)	Test Sample Total, n (%)
Total	174 (100)	98 (100)
Age		
Years (mean)	23.8	43.8
Gender		
Female	134 (77)	80 (82)
Male	38 (22)	16 (16)
No response	2 (1)	2 (2)
Race/Ethnicity		
African American	18 (10)	89 (91)
American Indian or Alaska Native	2 (1)	1 (1)
Asian	35 (20)	2 (2)
Hispanic	3 (2)	0 (0)
White	109 (63)	0 (0)
No response	3 (2)	6 (6)
Highest level of education		
Less than high school	0 (0)	4 (4)
High school diploma or equivalent	0 (0)	18 (18)
Post-secondary, non-degree	0 (0)	1 (1)
Associate degree	1 (1)	5 (5)
Some college, no degree	64 (37)	13 (13)
Bachelor’s degree	79 (45)	22 (22)
Master’s degree	25 (14)	25 (26)
Doctoral or professional degree	4 (2)	1 (1)
No response	1 (1)	9 (9)
Length of residence in current city		
Years (mean)	3.0	30.6
Taken an environmental health class		
Yes	133 (76)	35 (36)
No	40 (23)	57 (58)
No response	1 (1)	6 (6)

**Table 2:** Exploratory factor analysis results for the Air scale.

Items	K ^a^	A ^b^	B ^c^	Mean ± SD
Knowledge				
1. Storing chemicals like gasoline inside the home is not a problem as long as the container is closed.	0.64	0.04	0.01	2.86 ± 0.98
2. The air quality in my community is impacted by local industry.	0.38	0.34	−0.05	4.12 ± 0.87
3. Products that are used to freshen indoor air always improve indoor air quality	−0.65	−0.17	0.16	1.89 ± 0.72
Attitudes				
4. Indoor air pollution is not a problem in my state.	0.09	0.55	0.09	3.09 ± 0.98
5. Air pollution does not affect my or my family’s health.	0.09	0.41	0.02	3.11 ± 0.96
6. I consider the air I breathe in my community to be clean.	−0.07	−0.51	0.06	3.01 ± 0.8
Behaviors				
7. I have had my indoor air tested.	−0.1	−0.06	0.58	1.36 ± 0.86
8. I use face masks when cleaning my house.	−0.08	−0.04	0.5	1.75 ± 1.07
9. I avoid exercising because of pollution.	−0.03	−0.01	−0.4	3.61 ± 0.77
10. I avoid opening my window due to poor outdoor air quality.	0.03	−0.27	−0.45	2.99 ± 1.03

^a^ K = Knowledge, ^b^ A = Attitudes, ^c^ B = Behaviors.

**Table 3:** Exploratory and confirmatory factor analysis and Cronbach’s alpha for Air, Food, Water, and General environmental health scales.

Scale	EFA^a^ Fit	CFA^b^ Fit		Cronbach’s Alpha
	Absolute Index (X^2^)	Absolute Index (X^2^)	Parsimony Index ^c^	
Air	17.73; *p* = 0.47	41.23; *p* = 0.13	0.05	0.70
Food	16.55; *p* = 0.17	51.41; *p* = 0.001	0.11	0.67
Water	46.93; *p* = 0.67	119.77; *p* = 0.01	0.07	0.63
General	20.80; *p* = 0.29	28.56; *p* = 0.24	0.05	0.70

^a^ EFA = exploratory factor analysis, ^b^ CFA = confirmatory factor analysis, ^c^ Represents the root mean square error of approximation (RMSEA).

**Table 4:** Eigenvalues of retained factors for Air, Food, Water and General environmental health scales.

Scale	Knowledge	Attitudes	Behaviors
Air	2.27	0.79	1.42
Food	1.62	3.01	0.75
Water	2.54	1.35	3.19
General	1.66	1.30	4.75

**Table 5:** Exploratory factor analysis results for the Food scale.

Items	K ^a^	A ^b^	B ^c^	Mean ± SD
Knowledge				
1. Washing hands when making meals helps keep disease from spreading.	0.75	0.12	0.09	4.76 ± 0.48
2. Cutting a tomato on a cutting board after cutting raw meat without washing the board might lead to cross-contamination and spreading of disease.	0.57	−0.17	0.32	4.69 ± 0.65
Attitudes				
3. I believe that learning about food safety will benefit my health.	0.19	0.79	0.08	4.76 ± 0.48
4. I am willing to attend a food safety training course.	−0.13	0.65	0.06	3.55 ± 1.14
5. I select a place to purchase groceries based on its reputation and cleanliness.	−0.04	0.57	0.04	3.66 ± 1.14
6. I select a restaurant based on its reputation, cleanliness, and food safety score.	−0.05	0.55	0.04	3.58 ± 1.21
7. I am willing to change my food handling behaviors when I learn they are unsafe.	0.2	0.45	0.12	4.67 ± 0.61
Behaviors				
8. I use separate clean utensils to handle raw and fresh items while cooking.	0.12	0.11	0.99	4.40 ± 0.96
9. I use utensils to handle food that is ready to eat.	0.13	0.09	0.39	3.98 ± 0.90

^a^ K = Knowledge, ^b^ A = Attitudes, ^c^ B = Behaviors.

**Table 6:** Exploratory factor analysis results for the Water scale.

Items	K ^a^	A ^b^	B ^c^	Mean ± SD
Knowledge				
1. Chlorine is used to kill bacteria in water systems.	0.67	0.04	0.07	4.06 ± 0.86
2. Municipal (city) drinking water is processed at a water treatment facility before it is delivered to the public.	0.45	−0.13	0.03	4.09 ± 0.79
3. The government oversees the quality of the drinking water in cities around the country.	0.41	−0.06	0.03	3.51 ± 1.00
4. Shampoo and out of date medications flushed in the drain can be harmful to our water supply.	0.41	0.11	0.16	4.12 ± 0.91
Attitudes				
5. I often worry about safe drinking water.	−0.12	0.74	0.02	3.55 ± 1.19
6. I worry about chemicals in our drinking water.	0.01	0.64	0.01	3.90 ± 0.96
7. I worry about the quality of water because of old pipes in our homes.	−0.01	0.57	0.12	3.64 ± 1.19
Behaviors				
8. I only use the dishwasher when I have a full load.	0.08	−0.14	0.67	4.33 ± 1.11
9. I only wash clothes when I have a full load.	−0.04	−0.02	0.54	4.20 ± 0.97
10. I pay attention to how much time I spend in the shower in an effort to conserve water.	0.12	0.26	0.53	3.25 ± 1.3
11. I track water usage monthly using my water bill	−0.05	0.09	0.48	2.99 ± 1.52
12. I comply with instructions when a boil water advisory is issued by the city	0.17	0.0	0.4	4.48 ± 0.82
13. I turn off the tap water while brushing my teeth.	0.25	0.07	0.38	4.32 ± 1.03
14. I do not open the tap all the way while washing dishes.	−0.19	-0.22	−0.38	1.69 ± 1.28

^a^ K = Knowledge, ^b^ A = Attitudes, ^c^ B = Behaviors.

**Table 7:** Exploratory factor analysis results for the General environmental health scale.

Items	K ^a^	A ^b^	B ^c^	Mean ± SD
Knowledge				
1. Chemicals can be found in carpet, rugs, curtains, and furniture.	0.75	0.07	0.07	4.55 ± 0.61
2. Secondhand smoking is harmful to health.	0.40	−0.04	−0.09	4.96 ± 0.20
3. Cutting a tomato on a cutting board after cutting raw meat without washing the board might lead to cross-contamination and spreading of disease.	0.37	0.01	−0.21	4.70 ± 0.65
Attitudes				
4. I worry about the chemicals I am exposed to on a daily basis.	0.10	0.99	−0.04	3.14 ± 1.13
5. I worry about chemicals because they are always bad for my health.	−0.14	0.37	−0.06	3.36 ± 1.12
6. I think pollution is a problem, but there is nothing I can do to fix it.	0.06	0.26	−0.11	2.12 ± 0.85
Behaviors				
7. I avoid inhaling car exhaust.	−0.08	−0.05	0.85	4.16 ± 1.01
8. I avoid inhaling cleaning products.	−0.09	−0.14	0.73	3.86 ± 1.07
9. I avoid exposing myself and family members to harmful chemicals.	−0.11	−0.17	0.63	3.14 ± 1.13

^a^ K = Knowledge, ^b^ A = Attitudes, ^c^ B = Behaviors.
